# A Minimalist Approach With Maximum Outcomes: Intralesional Steroid Therapy in Orofacial Sarcoidosis

**DOI:** 10.7759/cureus.104145

**Published:** 2026-02-23

**Authors:** Muhammed Aseel Zahir Hussain, Sai Preethi P., Sudha Rangarajan, Leena Joseph, Adikrishnan S.

**Affiliations:** 1 Dermatology, Venereology and Leprosy, Sri Ramachandra Institute of Higher Education and Research, Chennai, IND; 2 Pathology, Sri Ramachandra Institute of Higher Education and Research, Chennai, IND

**Keywords:** intralesional corticosteroid, lip swelling, oral sarcoidosis, orofacial granulomatosis, sarcoidosis

## Abstract

Sarcoidosis is a multisystem granulomatous disease of unknown etiology, with oral cavity involvement being rare. Chronic orofacial swelling can mimic other conditions such as angioedema or orofacial granulomatosis (OFG), making diagnosis challenging.

We report the case of a 17-year-old male patient with progressive swelling of the lips and buccal mucosa since adolescence. Initially treated as angioedema with oral corticosteroids, he developed recurrent flares upon tapering. Histopathology from a lip biopsy revealed non-necrotizing granulomas consistent with sarcoidosis. Systemic disease was ruled out through imaging, serum angiotensin-converting enzyme (ACE) levels, and specialist evaluation. The patient was treated with three sessions of intralesional triamcinolone acetonide injections (40 mg/mL) at four-week intervals, with significant and sustained reduction in swelling over a nine-month follow-up period.

Oral sarcoidosis should be considered in chronic orofacial swelling. Intralesional corticosteroid therapy offers an effective and well-tolerated treatment, avoiding the systemic adverse effects of long-term oral steroids.

## Introduction

Sarcoidosis is a systemic granulomatous disorder characterized by non-caseating epithelioid granulomas, with pulmonary and lymphatic involvement being the most common manifestations. Oral involvement of sarcoidosis is rare and affects the jaw bone, buccal mucosa, lips, gingivae, tongue, and palate [1]. It can present with diffuse swelling, submucosal nodules that can occasionally either show superficial ulceration or be symptomatic while redness or enlargement can be the presentation in case of gingival involvement [2]. Early recognition is crucial to prevent misdiagnosis and inappropriate treatment.

## Case presentation

**Figure 1:**
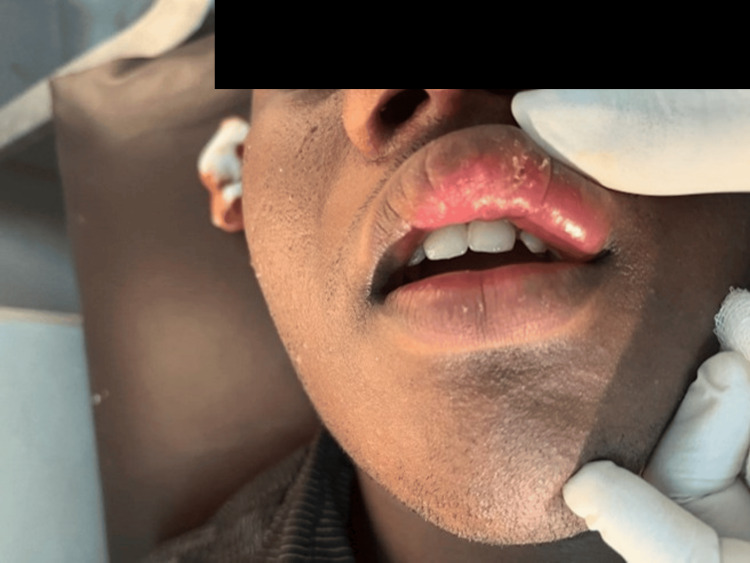
Swelling of upper lip prior to biopsy

**Figure 2:**
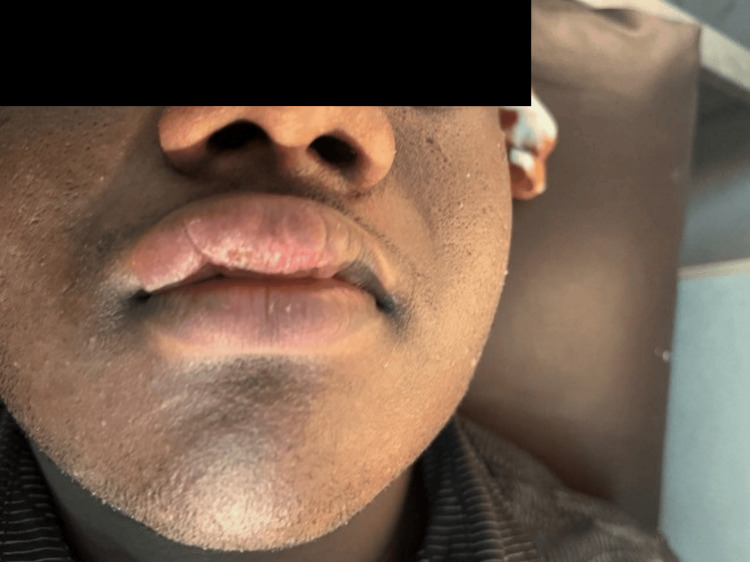
Swelling of lower lip prior to biopsy

A 17-year-old male patient presented with progressive swelling of the upper and lower lips since the age of 12, with subsequent involvement of the buccal mucosa (Figures 1, 2) .

Multiple courses of oral corticosteroids, given under the assumption of angioedema, led to temporary improvement but frequent relapses upon tapering. There was no history of urticaria, drug- or food-related aggravation, family history of angioedema, or swelling in other regions.

**Figure 3:**
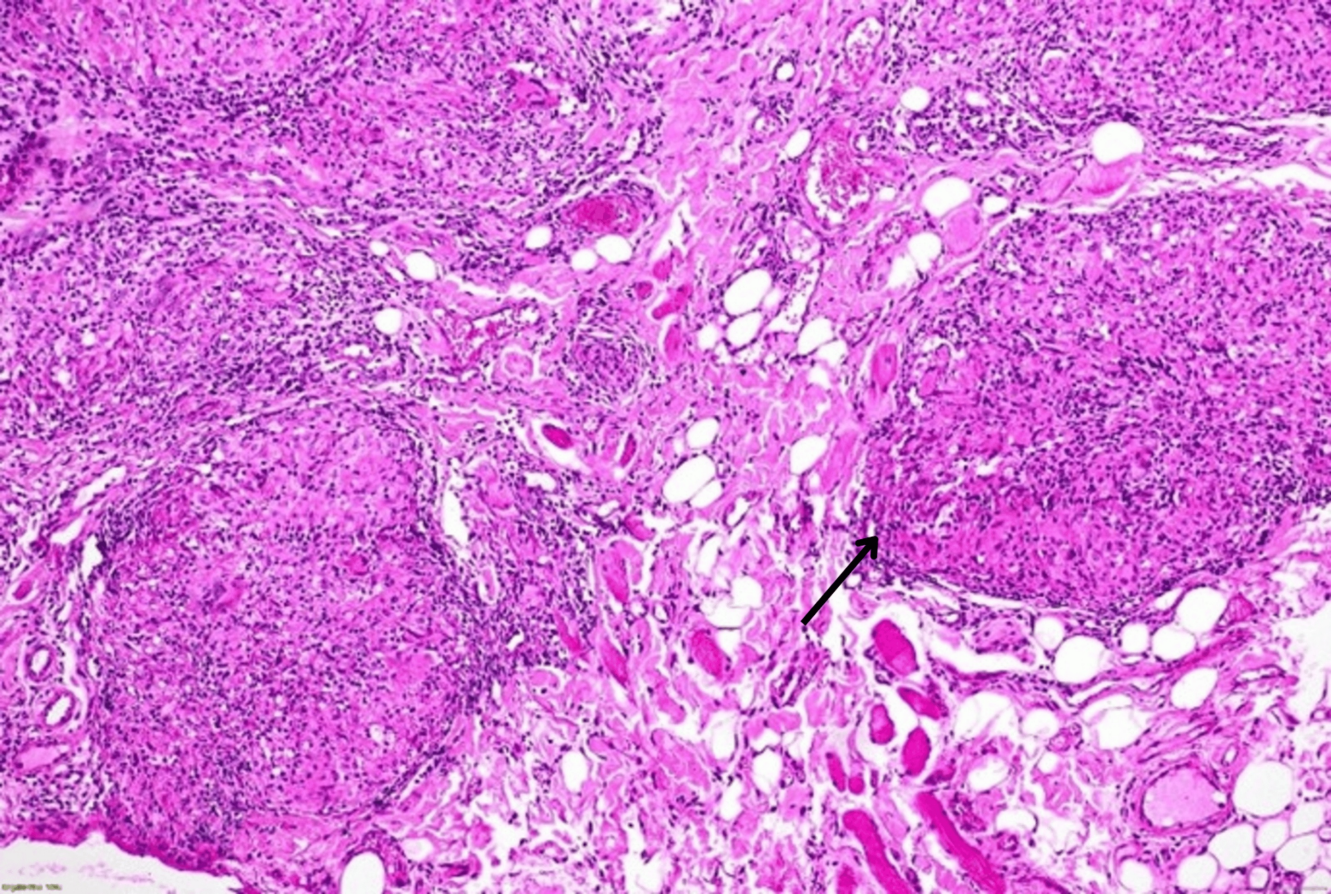
Histopathological examination (hematoxylin and eosin stain, 10x) - non-necrotizing epithelioid granulomas (black arrow)

Due to persistence, a 3 mm punch biopsy of the lower lip was performed. Histopathology showed non-necrotizing epithelioid granulomas with multinucleated giant cells extending into fat and muscle, consistent with sarcoidosis (Figure 3).

**Figure 4:**
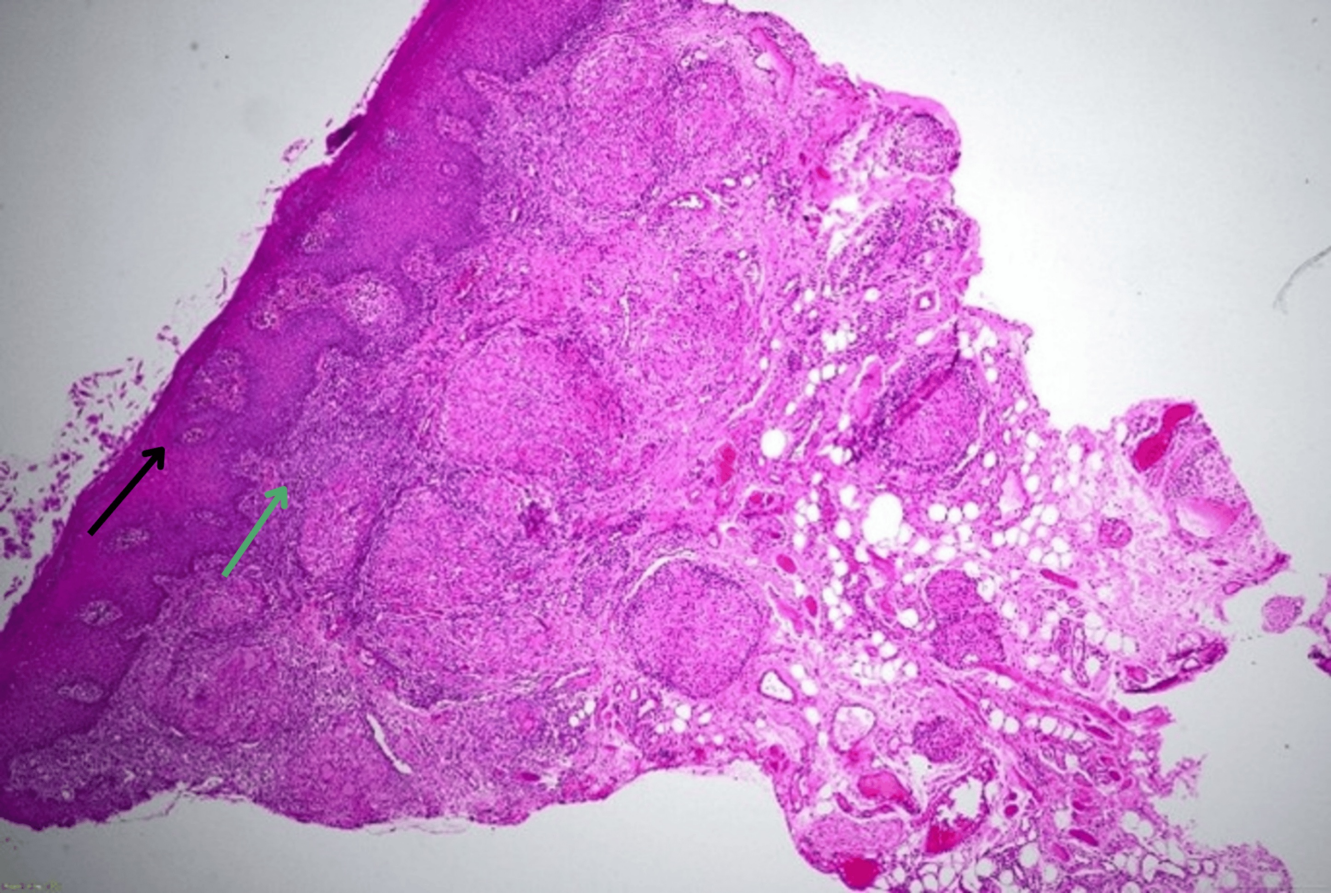
Histopathological examination (hematoxylin and eosin stain, 4x) showing epidermis with acanthosis, spongiosis, and parakeratosis (black arrow) with dense lymphoplasmacytic infiltrate at dermo-epidermal junction (green arrow)

The epithelium demonstrated acanthosis, spongiosis, and parakeratosis with a dense lymphoplasmacytic infiltrate at the dermo-epidermal junction (Figure 4).

Special stains (Wade-Fite) were negative for tuberculosis.

Serum angiotensin-converting enzyme (ACE) levels were normal. Chest radiography and pulmonology evaluation ruled out systemic sarcoidosis. Considering the side effects of prolonged systemic corticosteroid use, intralesional corticosteroid injections were initiated.

**Figure 5:**
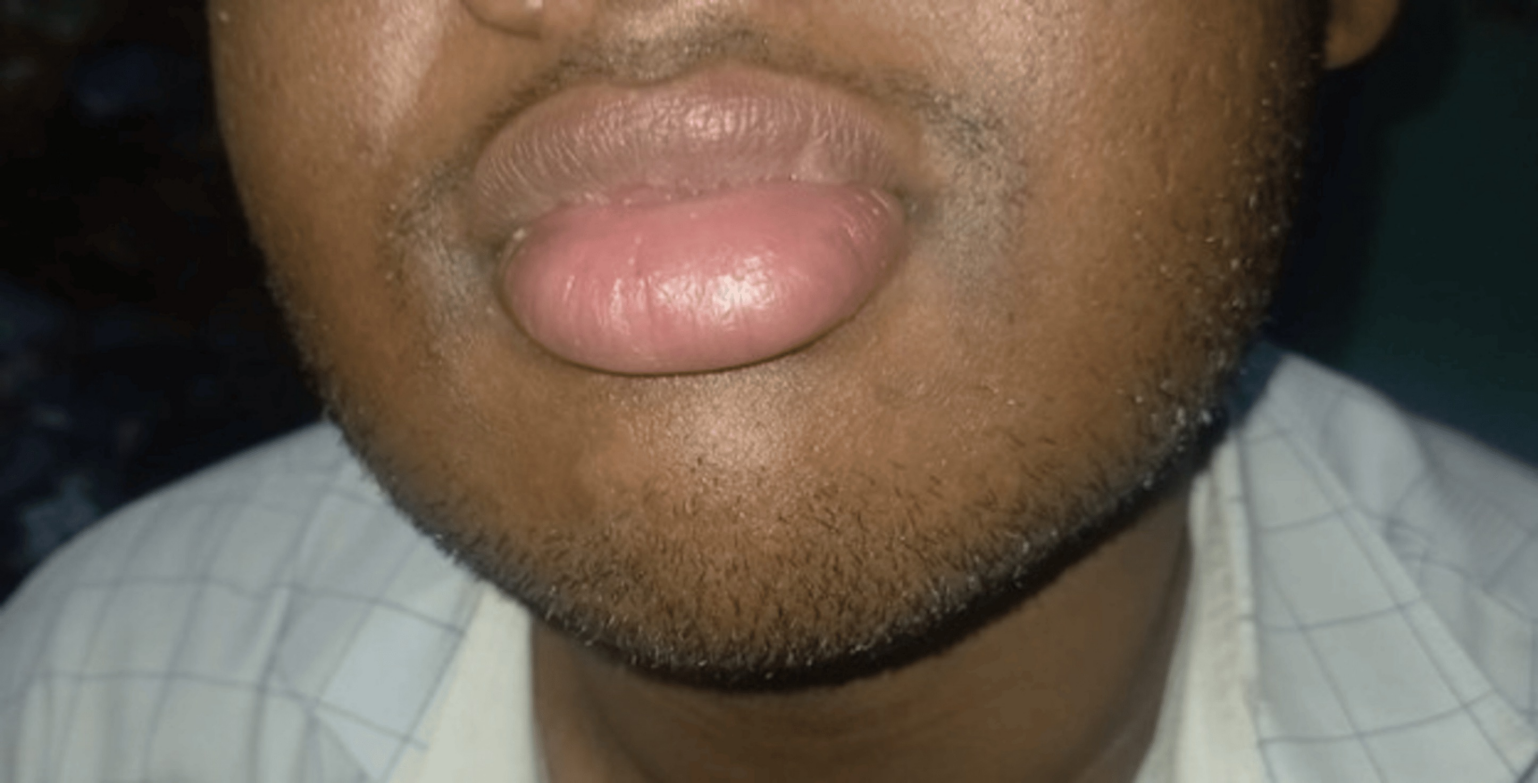
Absence of swelling of lip at nine-month follow up

The patient received three sessions of intralesional triamcinolone acetonide (40 mg/mL), administered at three-four sites per session, at four-week intervals. Progressive reduction in swelling was noted, and sustained remission was achieved following the last session in December 2025. No recurrence was observed during a nine-month follow-up (Figure 5) .

## Discussion

Sarcoidosis is a systemic non-caseating granulomatous inflammation with multifactorial etiology of environmental factor, infections, genetics or in many cases unknown etiology [3]. Common presentation includes nonspecific constitutional symptoms of fatigue, fever, and weight loss. Pulmonary involvement, hilar lymphadenopathy, and eye and skin manifestations are also commonly seen presentations. Medications like tumor necrosis factor (TNF) inhibitors, interleukin-7 (IL-7) inhibitors, and IL-1 receptor antagonist have been reported to induce cutaneous sarcoidosis or sarcoid-like granulomatous eruptions. Involvement of oral cavity is a rather uncommon manifestation with the buccal mucosa the commonest affected site at 30% followed by gingiva (20%), lips (16%), tongue (16%), and palate (9%) [3].

Oral sarcoidosis is an uncommon manifestation with buccal mucosa, gingiva, lips, and tongue being the most frequently affected sites. The differential diagnosis includes orofacial granulomatosis (OFG), Crohn’s disease, tuberculosis and immunoglobulin E (IgE)-mediated angioedema. 

Heerfordt-Waldenstrom syndrome manifests as facial palsy, parotid gland enlargement, and uveitis. Lofgren syndrome manifests as fever, bilateral hilar lymphadenopathy, and erythema nodosum. Immunological studies have shown association with CD4+ T cells, Th1 helper T cells, and macrophages [4].

OFG is characterized by non-necrotizing noninfectious granulomatous inflammation of face and mouth in absence of systemic disease. It is classified into primary and secondary. Primary OFG includes cheilitis granulomatosa (CG) and Melkersson-Rosenthal syndrome (MRS). Secondary OFG includes localized and systemic associations like sarcoidosis and Crohn's disease.

Oral sarcoidosis usually presents as intermittent swelling of upper or lower lips resembling angioedema progressing to a persistent swelling of the lips. The lack of remission with oral corticosteroid should alert the treating doctor to other causes for the lip swelling. Other labs like serum ACE levels and biopsy can be done to support the diagnosis. Serum ACE can be raised as it is produced by granulomas, but normal levels don’t rule out the disease [5]. Correlation has been noted between the granuloma burden and serum ACE levels. Serum ACE is helpful in determining the active status of sarcoidosis, and detection of the decrease in elevated blood ACE is a good marker for efficiency of therapy [6]. Histopathology remains the gold standard for diagnosis supported by exclusion of other causes. Histology shows dermal infiltrate of naked sarcoidal granuloma. Multiple epithelioid cell granuloma with varying degrees of necrosis, hyaline fibrosis, and infiltration by leukocyte can be seen [7]. Sparse lymphocytic infiltrate can be seen and because of this scarcity of lymphocytes, granulomas are referred to as “naked” tubercles. Granulomas present in superficial dermis or depending on type of cutaneous lesion can extend through dermis or subcutis. Once diagnosed as oral sarcoidosis, treatment becomes the challenging part due to frequent recurrence. The systemic agents used are nonsteroidal anti-inflammatory drugs (NSAIDs), broad-spectrum antibiotics, antituberculosis drugs, antilepromatous agents, antimalarials, sulfa drugs, and steroids. For resistant cases, especially when chronic inflammation is causing fibrous tissue proliferation, cheiloplasty has been suggested as it has shown some results. However, we opted for a simpler modality of treatment in our patient by treating him with intralesional steroid injections. Corticosteroids inhibit the downstream transcription of proinflammatory signal molecules like tumor necrosis factor-alpha (TNFα), granulocyte-macrophage colony stimulating factor (GM-CSF), and IL-1, 2, 3, 4, 5, 8, 11, and 13 by stimulating transcription of gene for inhibitor of NFκB (IκB) [8]. This is a treatment modality that improves patient adherence to treatment due to it being a walk-in treatment, requiring the patient to only attend his appointments on time; it’s a cost-effective treatment option; and it also reduces the possible side effects associated with systemic treatment with steroids. Side effects like hypopigmentation and atrophy of issue can be avoided with appropriate injection technique [9]. With it being a localized modality of treatment, the drug gets concentrated at the affected site as it can bypass the thickened stratum corneum and they have lesser side effects when contrasted with systemic corticosteroids [10]. As can also be seen from our experience, the patient achieved a nine-month remission with just three injections spaced four weeks apart, which proves the efficacy of this minimally invasive localized approach for treatment. Our experience has allowed us to think of intralesional corticosteroid as a first-line treatment modality even before offering systemic agents for the patient.

## Conclusions

Oral sarcoidosis, though rare, should be considered in the differential diagnosis of persistent orofacial swelling. A thorough systemic evaluation is essential to exclude multisystem involvement. Intralesional corticosteroid therapy is an effective and safe modality for localized disease, offering long-term control with minimal side effects.
